# Design and implementation of a custom next generation sequencing panel for selected vitamin D associated genes

**DOI:** 10.1186/s13104-017-2664-z

**Published:** 2017-07-28

**Authors:** Katherine Angela Benson, Sourabh Chand, Alexander Peter Maxwell, Laura Jane Smyth, Jill Kilner, Richard Borrows, Amy Jayne McKnight

**Affiliations:** 1 Nephrology Research Group, Centre for Public Health, Queen’s University Belfast, Belfast, BT9 7AB UK; 2 Department of Renal Medicine, Royal Shrewsbury Hospital, Shropshire, UK; 3 Department of Nephrology and Kidney Transplantation, Queen Elizabeth Hospital Birmingham, Birmingham, UK

**Keywords:** AmpliSeq, Association, Kidney, Next Generation Sequencing, NGS, Renal, Transplant, SNP, Vitamin D

## Abstract

**Background:**

Biologically active vitamin D has an important regulatory role within the genome. It binds the vitamin D receptor (VDR) in order to control the expression of a wide range of genes as well as interacting with the epigenome to modify chromatin and methylation status. Vitamin D deficiency is associated with several human diseases including end-stage renal disease.

**Methods:**

This article describes the design and testing of a custom, targeted next generation sequencing (NGS) panel for selected vitamin D associated genes. Sequencing runs were used to determine the effectiveness of the panel for variant calling, to compare efficiency and data across different sequencers, and to perform representative, proof of principle association analyses. These analyses were underpowered for significance testing. Amplicons were designed in two pools (163 and 166 fragments respectively) and used to sequence two cohorts of renal transplant recipients on the Ion Personal Genome Machine (PGM)™ and Ion S5™ XL desktop sequencers.

**Results:**

Coverage was provided for 43.8 kilobases across seven vitamin D associated genes (*CYP24A1, CUBN, VDR, GC, NADSYN1, CYP27B1, CYP2R1*) as well as 38 prioritised SNPs. Sequencing runs provided sufficient sequencing quality, data output and validated the effective library preparation and panel design.

**Conclusions:**

This novel, custom-designed, validated panel provides a fast, cost effective, and specific approach for the analysis of vitamin D associated genes in a wide range of patient cohorts. This article does not report results from a controlled health-care intervention.

**Electronic supplementary material:**

The online version of this article (doi:10.1186/s13104-017-2664-z) contains supplementary material, which is available to authorized users.

## Background

Biologically active vitamin D (1,25-dihydroxyvitamin D) is involved in the regulation of gastrointestinal calcium absorption and bone homeostasis [1]. A precursor (7-dehydrocholestrol) to active vitamin D is produced in the skin on exposure to ultraviolet B (UVB) radiation and is also found in certain foods such as oily fish and cheese [2]. Recent NICE guidelines recommend that pregnant and breastfeeding women, children ages 6 months to 5 years, adults over 65 years or anyone who is not regularly exposed to the sun should take a daily vitamin D3 (cholecalciferol) supplement up to 10,000 IU [3, 4]. A wide range of enzymes are involved in the metabolism and activity of vitamin D. Cytochrome P450 enzymes including CYP27B1, CYP2R1 and CYP24A1 contribute to the hydroxylation steps.

In the kidney, 1-α-hydroxylase, encoded by *CYP27B1* converts 25-hydroxyvitamin D3 to 1,25-dihydroxyvitamin D, directly affecting the circulating levels of active vitamin D. Polymorphisms within *CYP27B1* have previously been associated with the development of type 1 diabetes [5]. The *GC* gene encodes the vitamin D binding protein which has affinity for all vitamin D metabolites [6]. Polymorphisms within *GC* have been associated with reduced 25-hydroxyvitamin D3 levels [7].

The VDR is a steroid receptor expressed in more than 30 cell types [8]. VDR functions as a heterodimer with the retinoid X receptor (RXR) in the presence of vitamin D to activate transcription of vitamin D controlled genes [9, 10]. This is one of the mechanisms by which 1,25-dihydroxyvitamin D regulates the genome, but it has also been shown to affect the epigenome through chromatin modifiers and methylation changes [11]. It has been suggested that as much as 3% of the human genome is under some form of regulatory control by vitamin D [8, 12]. Two lymphoblastoid cell lines stimulated with 1,25-dihydroxyvitamin D were used to generate a ChIP-Seq genome-wide map, which identified 229 genomic regions differentially bound with VDR before and after treatment with active vitamin D [13]. Significant changes in gene expression after stimulation with 1,25-dihydroxyvitamin D were observed for gene loci such as *IRF8* and *PTPN2* which were not previously associated with vitamin D regulation [13]. Selected vitamin D related SNPs, within genes associated with vitamin D metabolism, are outlined in Table 1 [14–22].

**Table 1:** Genetic regions included in the custom AmpliSeq™ vitamin D panel

Type	Name	Chr	Amplicon start and end point	Amplicons	Coverage	Reason for missed coverage
Gene	*CUBN*	10		143	0.89059	
Gene	*CYP24A1*	20		28	0.9426	
Gene	*CYP27B1*	12		21	0.9049	
Gene	*CYP2R1*	11		14	1	
Gene	*GC*	4		27	0.8084	
Gene	*NADSYN1*	11		34	0.9829	
Gene	*VDR*	12		39	0.901	
SNP	rs10741657	11		0	0	Repetitive region
SNP	rs10875694	12		0	0	Repetitive region
SNP	rs11168275	12	48272163–48272406	1	1	
SNP	rs11168287	12	48285285–48285546	1	1	
SNP	rs11168314	12		0	0	Repetitive region
SNP	rs12721366	12	48245439–48245713	1	1	
SNP	rs12785878	11		1	1	
SNP	rs12794714	11		0	1	
SNP	rs1544410	12	48239730–48239989	1	1	
SNP	rs1989969	12	48277824–48278036	1	1	
SNP	rs2060793	11		0	0	Repetitive region
SNP	rs2071358	12	48366302–48366575	1	1	
SNP	rs2107301	12	48255540–48255664	1	1	
SNP	rs2189480	12	48263731–48263939	1	1	
SNP	rs2228570	12	48272752–48272975	1	1	
SNP	rs2238135	12	48278010–48278284	1	1	
SNP	rs2238139	12	48256146–48256419	1	1	
SNP	rs2239181	12	48255772–48256045	1	1	
SNP	rs2239182	12		0	0	Repetitive region
SNP	rs2298850	4	72614158–72614402	1	1	
SNP	rs2408876	12	48273431–48273704	1	1	
SNP	rs2544038	12	48215081–48215350	1	1	
SNP	rs2544043	12		0	0	Repetitive region
SNP	rs3740165	10		0	1	
SNP	rs3782905	12	48266129–48266327	1	1	
SNP	rs3922882	12	48284784–48284997	1	1	
SNP	rs3923693	12	48337491–48337729	1	1	
SNP	rs4073729	12	48337000–48337176	1	1	
SNP	rs4303288	12	48336602–48336727	1	0.5	Repetitive region
SNP	rs4646536	12		0	1	
SNP	rs4760674	12		0	0	Repetitive region
SNP	rs6013897	20		0	0	Repetitive region
SNP	rs6823	12	48362278–48362509	1	1	
SNP	rs7299460	12	48296145–48296402	1	1	
SNP	rs731236	12	48238644–48238859	1	1	
SNP	rs739837	12	48238171–48238400	1	1	
SNP	rs7974708	12		1	1	
SNP	rs7975232	12		1	1	

This table describes the genomic regions targeted by the custom vitamin D panel

Although the cost of both whole genome and exome sequencing is falling, these technologies are not always cost effective for routine use in laboratories or large-scale population studies. The bioinformatic analysis and raw data storage space required for large scale sequencing projects is often prohibitive. As an alternative, researchers may opt to sequence targeted genetic regions using sequencing panels [23]. There are two central approaches to enrich for targeted DNA regions; solution hybridisation using oligo-nucleotide probes such as the Agilent SureSelect™ custom panels, or PCR enrichment such as Ion Torrent AmpliSeq™ panels. During this study an Ion AmpliSeq™ (http://www.ampliseq.com) customised panel was designed using the Ion AmpliSeq™ Designer online tool and primer pairs to enrich genomic regions known to be associated with vitamin D [14, 15]. This PCR enrichment method was used as it provides deep and even coverage over the small genomic regions of interest which were targeted in our study and delivers higher rates of on-target sequencing [24, 25]. In contrast, where larger regions such as the whole exome are being targeted, hybridisation methods are recognised to be more suitable due to a higher rate of uniformity [25]. A comprehensive list of all regions targeted by the panel is included in Table 1.

The Ion PGM™ desktop sequencer, released in 2010, is ideally suited to sequencing selected batches of amplicons such as those used in AmpliSeq™ sequencing panels [26]. Increases in sequencing throughput between the Ion 314™, Ion 316™ or Ion 318™ silicon chips used by the Ion PGM™ are due to differences in chip size and closer packing of wells. The Ion S5™ and Ion S5™ XL sequencers were released in 2015, offering increased speed of data analysis, new scalable sequencing chips and considerably reduced hands-on-time. The Ion S5™ XL uses Ion 520™, Ion 530™ and Ion 540™ chips. It has a larger processor and is therefore capable of producing results faster and with a higher throughput than the Ion S5™. Sequencing output using Ion Torrent sequencers will vary depending on the input material, read lengths, chemistry employed, and the type of chip used.

Vitamin D deficiency has been suggested to be a risk factor for chronic kidney disease (CKD) [27–29]. A meta-analysis conducted in 2011 showed that higher vitamin D levels are associated with increased survival in CKD patients [30]. Increased activity of the renin-angiotensin system is strongly associated with CKD and diabetes [31]; vitamin D is a negative regulator of the renin-angiotensin system [12].

Many kidney transplant centres in the United Kingdom (UK) routinely prescribe vitamin D supplements for kidney transplant recipients [32]. The kidney transplant population is prone to vitamin D deficiency due to several factors including avoidance of direct sunlight, dietary deficiency, renal impairment (limiting the activation of vitamin D because of reduced 1-alpha hydroxylation) and anti-rejection medication such as corticosteroids which are known to increase metabolism of vitamin D. Kidney transplant patients have an increased risk of skin cancers and so are encouraged to avoid direct sunlight, thereby reducing the vitamin D which these patients produce in response to UVB exposure [33]. A 2007 UK study which tracked vitamin D levels in 104 newly transplanted and 140 long-term renal transplant recipients found that vitamin D deficiency was present in the majority of investigated patients [34]. Existing literature shows that vitamin D deficiency also increases risk of the development of the common renal transplant complication, new onset diabetes after transplantation (NODAT) [35, 36]. Vitamin D is known to increase insulin sensitivity and deficiency of vitamin D may also aggravate transplant-related insulin resistance [37]. A recent study by Keyzer and colleagues which included 435 stable renal transplant recipients demonstrated that low vitamin D levels were independently associated with all-cause mortality and a more rapid decline in eGFR over time [38].

The aim of our study was to design, validate and apply a custom Ion AmpliSeq™ NGS panel targeting selected vitamin D associated genetic regions. As described, vitamin D deficiency is strongly associated with chronic kidney disease and NODAT. Therefore, the panel was tested in two renal transplant populations to demonstrate its efficacy and results compared between two sequencers.

## Methods

Blood derived DNA was used for this study which was stored at −20 °C. DNA was extracted using the salting out method. The cohorts used for testing of the panel were: (1) a cohort of kidney transplant recipients (n = 77) from Belfast, Northern Ireland; (2) kidney transplant patients (n = 93) in a Birmingham-based cohort who had vitamin D levels and oral glucose tolerance test (OGTT) results measured prospectively at three separate time points (immediately before transplant, 3 months post-transplant and 12 months post-transplant) [39]. The patient characteristics for each cohort are summarised in Table 2. Vitamin D levels were measured in batches at an accredited NHS hospital lab from samples frozen at −40 °C, using mass spectrometry for serum total 25-hydroxyvitamin D. Possible confounders for vitamin D levels such as the season on blood sampling, skin colour, extensive use of high protection sunscreens or covering with clothing were not accounted for in this study.

**Table 2:** Patient cohort characteristics

Cohort	Belfast	Birmingham vitamin D analysis	Birmingham NODAT replication
Total individuals (n)	77	93	70
NODAT			
Cases [n(%)]	33 (43%)	18 (19%)	16 (23%)
Controls [n(%)]	44 (57%)	75 (81%)	54 (77%)
Average age in years (range)	45 (17–71)	44 (17–71)	46 (17–71)
Sex			
Male [n(%)]	50 (65%)	56 (60%)	44 (63%)
Female [n(%)]	27 (35%)	37 (40%)	26 (37%)
Average weight (kg) (range)	112 (82–164)	77 (49–132)	79 (50–132)
Race	Caucasian	Mixed ethnicity	Caucasian
# Blood collections	1	3	3

The Belfast cohort was sequenced using both the Ion PGM™ and the Ion S5™ XL sequencers in order to compare variant calling efficiency and technical sample preparation. Variants called differently between sequencers were further investigated using Sanger sequencing to determine which next generation sequencer had identified the variant correctly. In addition, an applied association analysis was undertaken to determine if the targeted variants were associated with vitamin D levels or NODAT in each of these cohorts.

At the time of listing for transplantation, all potential kidney transplant recipients were asked for written informed consent (or consent from a parent or guardian in the case of children) for their data to be stored within the Kidney Transplant Database and used in projects in an anonymised form. Approval for use of this database was granted by Office for Research Ethics Committees Northern Ireland (ORECNI)—reference number ORECNI 12/NI/0178.

A total of 77 transplant recipients were included in this study from the Belfast renal transplant cohort. NODAT was defined in this cohort as the new requirement for oral hypoglycaemic agents or insulin as a result of post-transplant hyperglycaemia. The average age of the included patients was 45 with an age range of 12–71 years. There were more male renal transplant recipients (n = 50) than female recipients (n = 27) which is consistent with the established statistics that men are more likely to develop end stage renal disease (ESRD) than women [40]. The Belfast cohort patients included in this study were of white ethnicity. The Birmingham renal transplant cohort is more ethnically diverse and the patients included in this study reflect this. The average age of patients in this group was 44 years with an age range of 17–71 years. NODAT was defined in the Birmingham cohort if (a) fasting glucose ≥7 mmol/L or 2 h OGTT was ≥11.1 mmol/L from day 7 onwards and persisted at the 3 month timepoint, (b) HbA1c ≥6.5% (48 mmol/mol) from 3 months onwards, or (c) requirement for institution of therapy for NODAT in which case OGTT was not undertaken (fasting clinic glucose was ≥7 mmol/L in all such patients). Seventy Caucasian patients with recorded data on NODAT status were included as a replication cohort for the Belfast group NODAT association analysis.

The custom vitamin D panel targeting genes (Table 1) and SNPs associated with vitamin D identified during previous research with collaborators in Birmingham was designed using the Ion AmpliSeq™ Designer™ online tool [14, 15]. Four of these targeted genes were chosen after their reported association with vitamin D insufficiency in a genome-wide association study published in the Lancet in 2010 (*GC, NADSYN1, CYP2R1, and CYP24A1*) [7]. In addition, a list of six genes which code for proteins which are established components of vitamin D metabolism (*CYP27A1, GC, CYP2R1, VDR, CYP27B1 and CYP24A1*) were chosen in light of a publication by Cooper and colleagues in 2011 [41]. This paper discussed inherited variation in vitamin D genes and their association with predisposition to type 1 diabetes [41]. These prior publications informed the list of seven genes included in the panel. A previous study by Nejentsev and colleagues identified *VDR* polymorphisms which are necessary to study common variation in populations from the British Isles; these important SNPs were also included in the panel [42]. The four classically genotyped SNPs (BsmI, FokI, TaqI, ApaI) were also contained within the panel for completeness. Further SNPs were selected from previous experimental work from our group completed in collaboration with colleagues from Birmingham [14]. As part of this research, in which the linkage disequilibrium structure for vitamin D in a UK population and additional ancestries was elucidated, robust sequencing methods including Sequenom and Sanger sequencing were employed [14]. These regions include large and small genes and are representative of other regions of the genome which groups which may use the Ion AmpliSeq™ method to sequence. These chosen targets (including coding and untranslated regions and 50 bp exon region flanking for genes) were entered into the online tool to generate BED files. The resulting amplicons were divided by the online designer into two primer pools to maximise target specificity.

These experiments employed early access reagents for the Ion S5™XL System, and Ion AmpliSeq™ technology, from Thermo Fisher Scientific. Libraries were prepared using the Ion AmpliSeq™ library kit 2.0 according to the manufacturer’s instructions. Genomic DNA (20 ng/sample) was used for the initial PCR reactions. Samples were diluted no more than 4 h prior to sequencing to prevent DNA degradation. The Ion Chef™ or Ion One Touch™ and ES™ were used for emulsion PCR and target enrichment. The Ion PGM™ was used with Ion 318 V2™ or Ion 316 V2™ chips to sequence the renal transplant cohorts. The Belfast kidney transplant recipients were re-sequenced using the Ion S5™ XL sequencer on Ion 530™ chips. Reported SNPs from the Ion Torrent sequencing runs on the Belfast kidney transplant cohort were compared between the two next generation sequencers. Variants found on one sequencer, but not the other, were then re-sequenced using Sanger sequencing. If possible, a sample which was reported with the variant on both sequencers, a sample which was reported as not having the variant on both sequencers and the sample in which the discrepancy was identified were also Sanger sequenced.

**Fig. 1:**
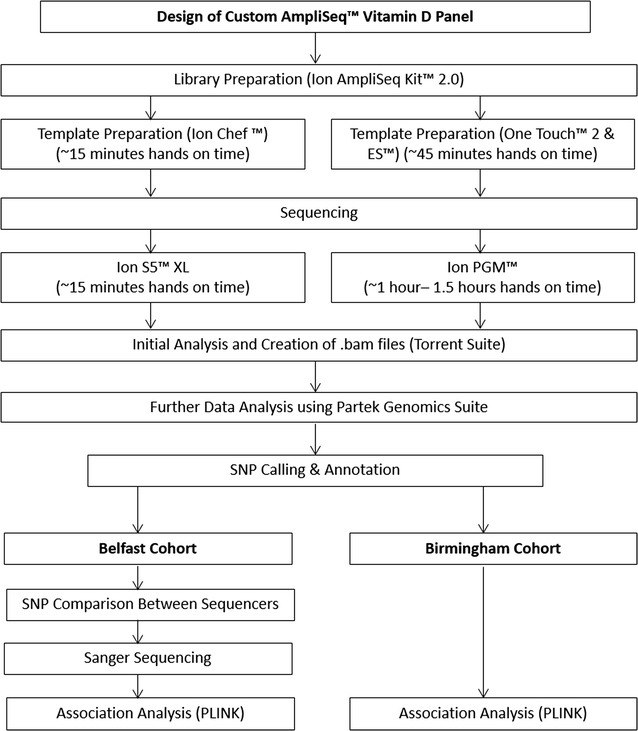
Overview of data analysis workflow. This image provides an overview of the described laboratory and analyses processes

An overview of the analysis workflow is illustrated in Fig. 1. Preliminary analysis was conducted automatically on the Ion Torrent Suite™ Version 4 where data was aligned to hg19.p5. Variant caller files generated from this analysis were used to compare the results between the Ion PGM™ and Ion S5™ XL sequencers. Sequencing data in the form of.bam files were further analysed using Partek Genomics Suite™. SNPs were identified using dbSNP Version 138 and annotated using RefSeq Version 2015-08-04. The resulting variant files from Partek Genomics Suite™ were used for association analysis.

To complete the association analysis,.ped files were generated for use with PLINK software Version 1.07. An unadjusted genotype association test for trend was used to find associations between NODAT status and the targeted variants in the Belfast renal transplant cohort using a *P* value threshold of 0.05. This *P* value is reported in this manuscript as *P*_*trend*_ Variants with Hardy–Weinberg equilibrium (HWE) <1 × 10^−5^ were removed from the analysis. In previous studies, transplant recipient age and body mass index (BMI) were shown to be important risk factors for the development of NODAT [43–46]; these variables were included in the regression model (*P*_*LR*_). Changes in vitamin D levels at 3 and 12 months after transplant were evaluated for association with gene variants in the Birmingham kidney transplant cohort regarding vitamin D levels post-transplant as a quantitative trait (*P*_*trend*_). As with the association analyses in the Belfast cohort, variants with a minor allele frequency (MAF) of <0.05 or a Hardy–Weinberg equilibrium (HWE) <1 × 10^−5^ were removed from the analysis. Recipient age and weight at transplant was included in this regression model (*P*_*LR*_). In addition, the association between NODAT and the identified gene variants was investigated in a subset of the Birmingham cohort (n = 70). All modelling assumed an additive inheritance pattern.

Sanger sequencing data was analysed using Vector NTI Advance™ Version 11.5.1. The resulting data files were aligned using Contig Express™ against the GRCh38 reference genome obtained from the Ensembl online resource (Version 8.4) [47]. The resulting chromatograms were visually inspected to determine whether the variant was identified correctly using NGS.

## Results

Targeted SNPs, along with translated and untranslated regions for seven genes associated with vitamin D regulation were successfully included in the custom panel (90.58% total coverage; 43.76 kb; Table 1). The targeted SNPs were shown in previous publications to be important in Caucasian populations for vitamin D metabolism and regulation and were located within the seven chosen genes [42]. Amplicons were automatically split between two pools containing 163 and 166 amplicons. These individual amplicons were 124–274 bp in length with an average size of 207 bp. PCR primers used in Ion AmpliSeq™ kits provided a high level of specificity and simplicity.

**Fig. 2:**
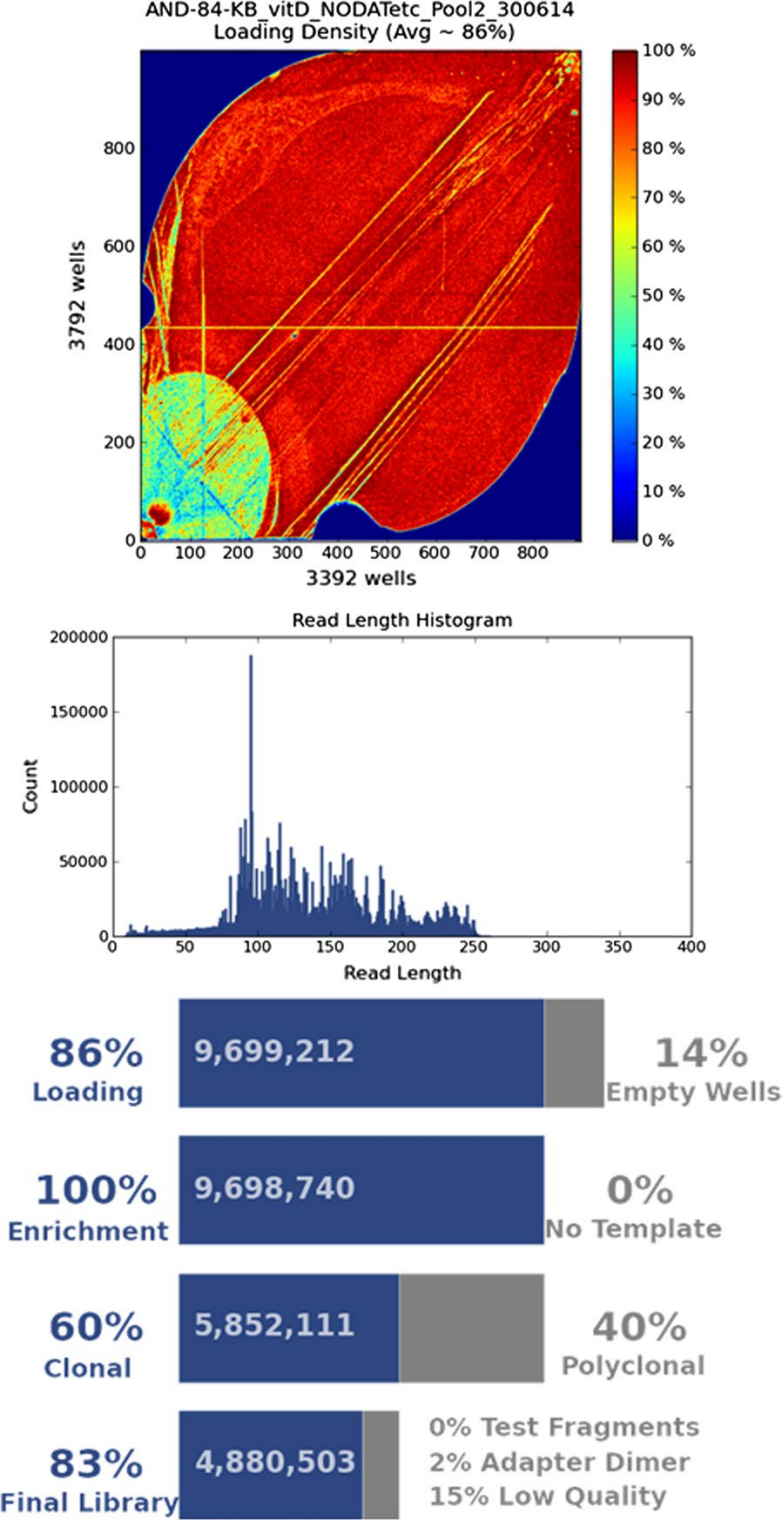
Sequencing statistics from an Ion PGM™ sequencing run. This figure demonstrates the loading density, read length histogram and summary statistics obtained from the Ion Torrent Suite™ following a representative Ion PGM™ sequencing run

**Fig. 3:**
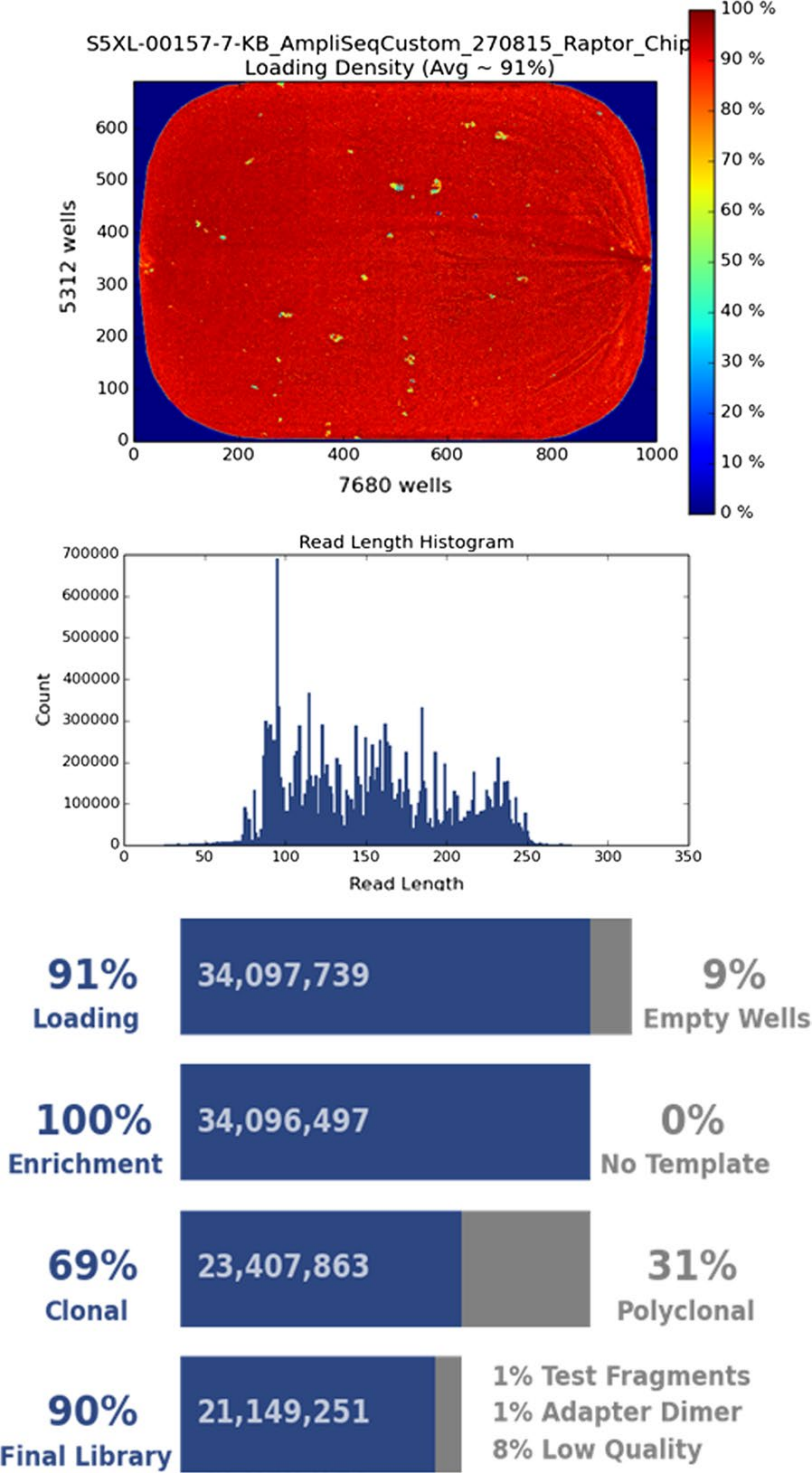
Sequencing statistics from an Ion S5™ XL sequencing run. This figure demonstrates the loading density, read length histogram and summary statistics obtained from the Ion Torrent Suite™ following a representative Ion S5™ XL sequencing run

Analysis on the Ion Torrent Suite™ included determination of the Ion Sphere Particle (ISP) loading, the number of usable reads and the length of sequencing reads for each run. Figures 2 and 3 show representative sequencing statistics for sequencing runs performed on the Ion PGM™ and Ion S5™ XL sequencers respectively.

**Fig. 4:**
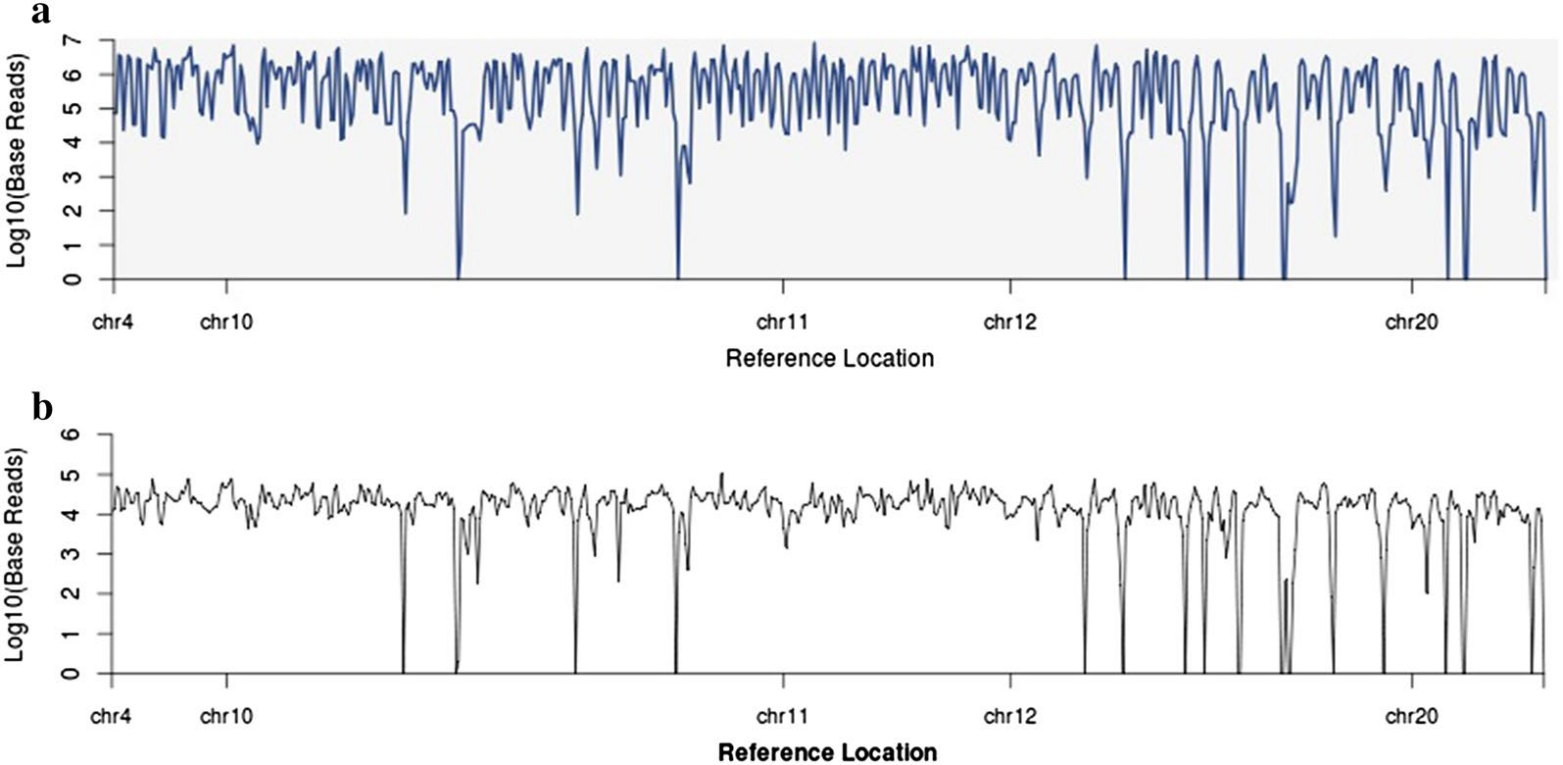
Representative coverage plots from **a** Ion S5™ XL and **b** Ion PGM™ sequencing runs. Both **a** and **b** show the consistent, high coverage levels obtained from both Ion S5™ XL and Ion PGM™ sequencing runs respectively. The Ion S5™ XL obtained slightly higher sequencing coverage (A)

Additional analysis was performed using plug-ins including variant caller, coverage analysis, and file exporter. Coverage was calculated using the coverage analysis plug-in, and representative plots are shown in Fig. 4. The hands on time required for the Ion PGM™ (approximately 1–1.5 h) was considerably higher than for the Ion S5™ XL (approximately 15 min) (Fig. 1).

A summary of the maximum output statistics for the sequencing chips from the NGS runs completed on the Ion PGM™ and Ion S5™ XL are shown in Table 3. Nine of the 11 sequencing runs were completed on the Ion PGM™ and two were completed on the Ion S5™ XL. Similar percentages of usable reads were obtained from all comparable runs. Polyclonal levels varied depending on the dilution factor used following library preparation and had a marked impact on the percentage of usable reads. These polyclonal levels increased with respect to the level of DNA input. The sequencing output varied from 429 Mb to 3.22 Gb and the total number of reads was relatively consistent ranging from 2,755,634 to 80,308,654. These data obtained for the two different cohorts were comparable although those obtained using the Ion PGM™ were significantly lower than those obtained using the Ion S5™ XL.

**Table 3:** Sequencing runs completed using both the Ion PGM™ and Ion S5™ XL

Sequencing Runs per chip								
Sequencer	Chip type	Dilution (pM)	Preparation	Max. output	Samples	Usable reads (%)	Max. reads	Polyclonal (%)
Ion PGM™	Ion 316™	26	Ion Chef™	429 Mb	8–12	51–57	2,755,634	21–37
Ion PGM™	Ion 318™	26/50	Ion Chef™/Manual	668 Mb	48–96	48–50	4,880,500	11–40
Ion S5™ XL	Ion 530™	50	Ion Chef™	3.22 Gb	85	50–61	80,308,654	26–30

Libraries were prepared using 20 ng genomic DNA. Dilution factors chosen for the sequencing runs were either 26 or 50 pM depending on the number of samples to be sequenced and the level of coverage desired

Based on the maximum output achieved by each of the different chip types used in this study, we observed a 1.6 fold increase between data output from the Ion 316™ and Ion 318™ sequencing chips and a 4.8 fold increase between the Ion 318™ and Ion 530™ sequencing chips (Table 3).

**Fig. 5:**
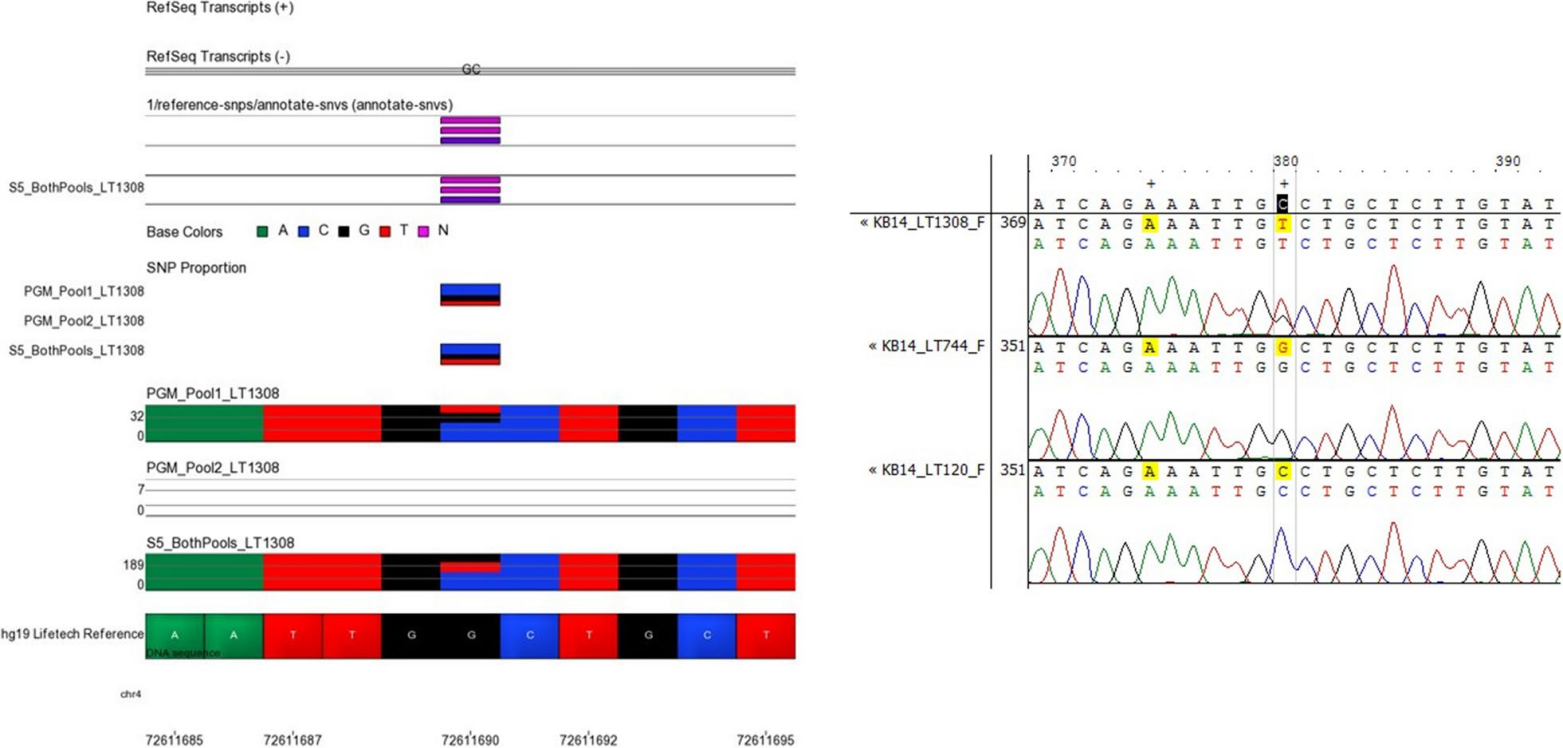
NGS and Sanger sequencing results at chromosome 4, location 72611690. A tri-allelic variant was identified at this locus. The variant was reported by the Ion S5™ XL variant caller file but not the Ion PGM™ variant caller file despite appearing in both in Partek Genomics Suite™ (on *left*). Sanger sequencing on the *right* for sample LT1308 as well as two other samples (LT744, LT120) shows that sample LT1308 is a T/C heterozygote while sample LT744 is G/G and sample LT120 is C/C

**Fig. 6:**
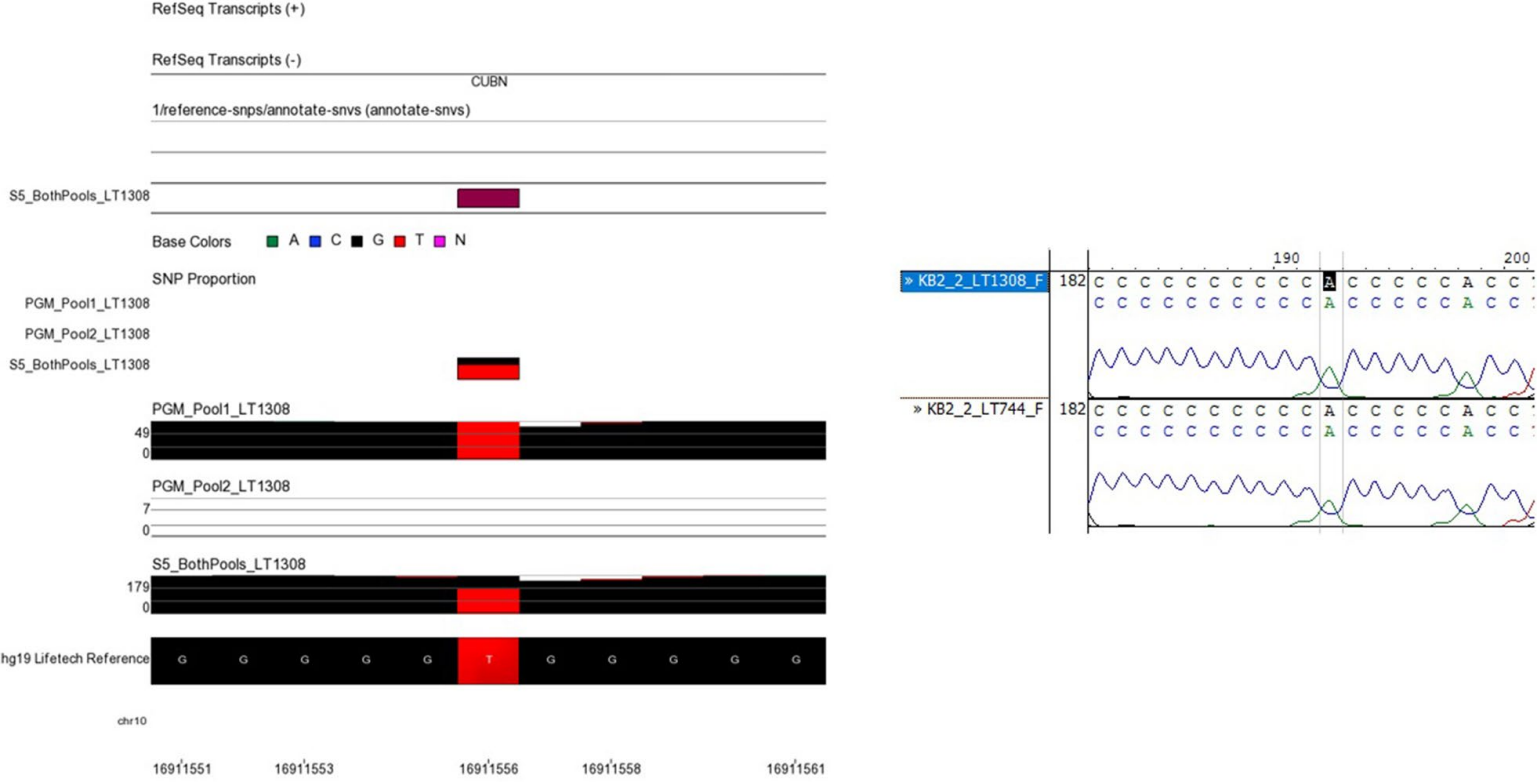
NGS and Sanger sequencing results at chromosome 10, location 16911556. At this locus, a heterozygote G/T variant was called in sample LT1308 by the Ion S5™ XL but not the Ion PGM™. Visual inspection of the Sanger sequencing results (shown here on the reverse strand) determined that the variant had been called incorrectly. There is a peak shown for A which matches the reference sequence. It is likely that the homo-polymer region was the reason the Ion S5™ XL had difficulty reporting this SNP

**Fig. 7:**
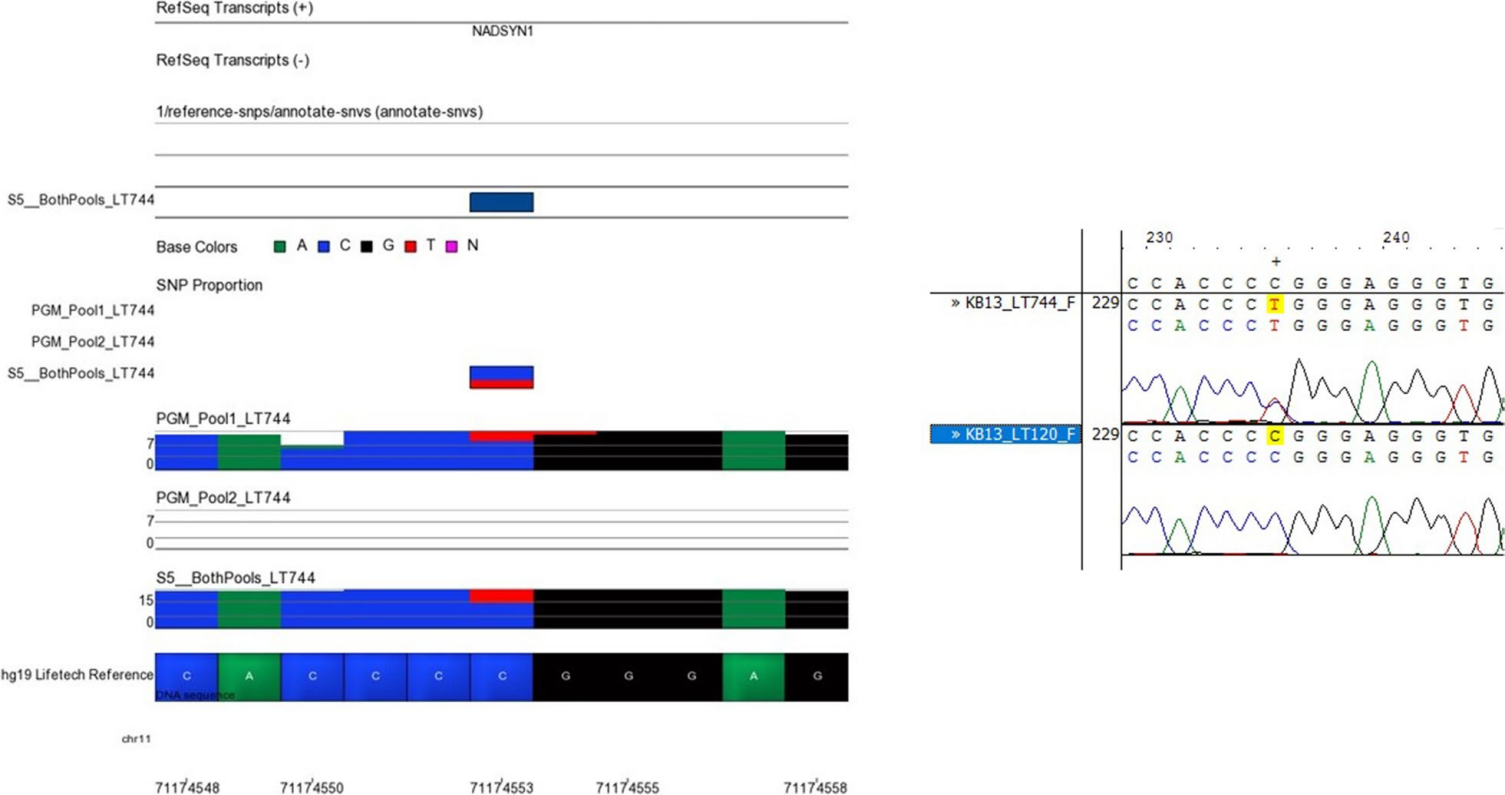
NGS and Sanger sequencing results at chromosome 11, location 71174553. Sample LT744 shown in Partek on the *left* of this figure, was reported to have a heterozygous C/T SNP at position chr11:71174553. This was detected by the Ion S5™ XL but not the Ion PGM™. Sanger sequencing results shown on the *right* of the figure confirmed that sample LT744 has a C/T SNP at this locus which is not present in sample LT120

**Fig. 8:**
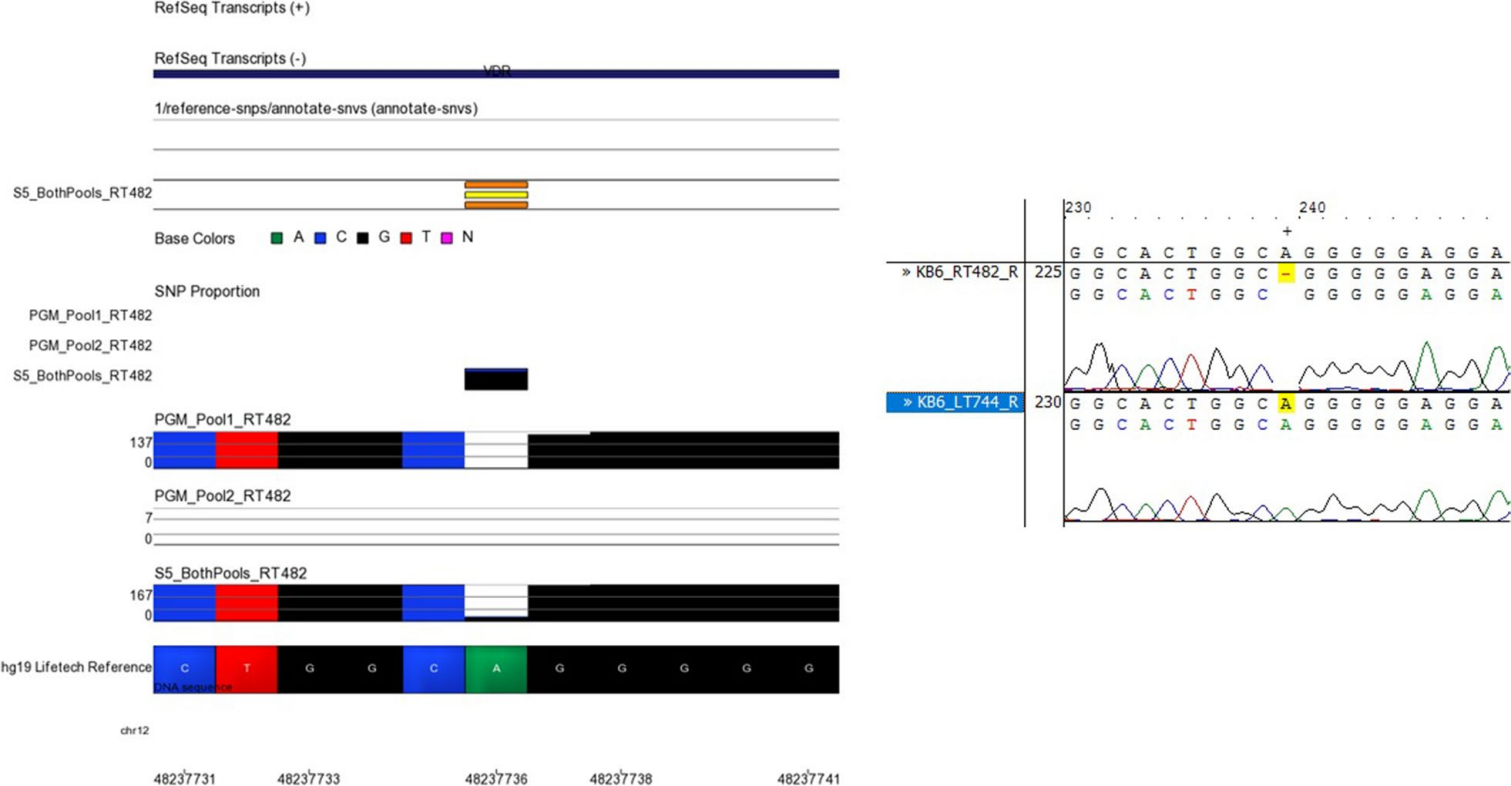
NGS and Sanger sequencing results at chromosome 12, location 48237736. The reference genome here shows an A base at locus chr12:48237736. The Ion S5™ XL reported a G/G variant. Sanger sequencing confirmed that there was a deletion at this locus in sample RT482 followed by five G bases. This deletion is not shown in sample LT744 which is also shown in the Sanger sequencing results on the *right* of this figure

**Fig. 9:**
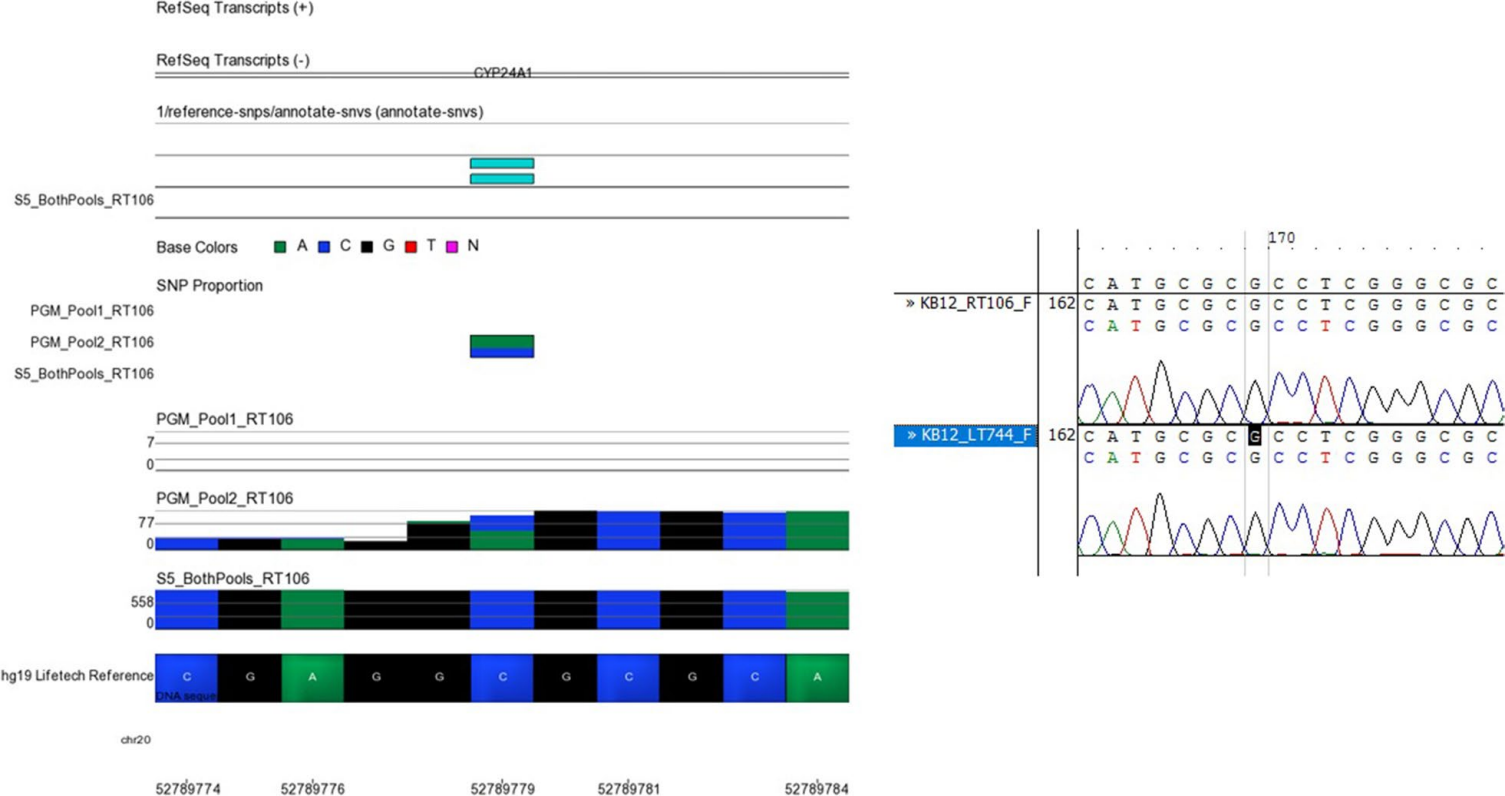
NGS and Sanger sequencing results at chromosome 20, location 52789779. The Partek figure on the left shows a C/A variant in sample RT106 identified in pool 2 of the Ion PGM™ sequencing results. This was not called by the Ion S5™ XL. Sanger sequencing on the right confirmed that there is no variant at this locus in either sample RT106 or sample LT744. A single peak is shown for C in both of these samples (the Sanger sequencing image to the *right* of this image is shown in the reverse complement). These results confirm that the Ion PGM™ called this variant incorrectly

Libraries for the Belfast renal transplant cohort were sequenced on both the Ion PGM™ and Ion S5™ XL. Due to the higher capacity of the Ion 530™ chip both amplicon pools were combined on the same sequencing chips for the Ion S5™ XL sequencing runs. Twenty-three SNPs were identified from the variant caller plug-in on the Ion Torrent Suite™ on one sequencer but not the other. Sanger capillary sequencing was used to determine which SNPs were genuine using 14 sets of PCR primers. Sanger sequencing is regarded as the gold standard for variant identification [48]. The SNP locations in samples with and without the proposed variants were visually inspected using Contig Express™. The Ion S5™ XL correctly identified nucleotide bases more often than the Ion PGM™ at these discordant SNP calls locations. Representative examples of the visual inspection of these SNPs using Partek Genomics Suite™ for NGS results and Contig Express™ for Sanger sequencing results are shown in Figs. 5, 6, 7, 8, 9. The alleles with ambiguous calls were identified correctly prior to association analysis.

The data obtained from the sequencing runs outlined in Table 3 was analysed on Partek Genomics Suite™ and used to perform association analysis to demonstrate the efficacy of the custom panel. Following analysis on Partek Genomics Suite™, off target and low coverage (less than 30 fold) SNPs were removed along with SNPs identified as incorrect following Sanger sequencing.

Initially, the association between development of NODAT post-renal transplant and the vitamin D variants in the panel was investigated in the Belfast kidney transplant population. In total, 457 SNPs were included for analysis from sequencing data obtained using the Ion S5™ XL. The top hits from this genotype association analysis using a nominal significance threshold of *P*_*trend*_ < 0.05 along with the corresponding results of the regression analyses are shown in Additional file 1: Table S1.

The association between NODAT development and the target variants was also investigated in a subset of the Birmingham kidney transplant cohort (n = 70) which included 16 NODAT cases and 54 renal transplant controls. The top hits are shown in Additional file 1: Table S2 along with the results of the regression analysis. In total, nine variants were nominally associated with NODAT development in the Birmingham cohort at the *P*_*trend*_ < 0.05 significance threshold in the genotypic test for trend. One variant was nominally associated with NODAT status in both the Belfast and Birmingham cohorts (rs1801239).

The association between the changes in vitamin D levels 3 or 12 months following renal transplant and the variants targeted on the custom panel were investigated in the Birmingham kidney transplant cohort (n = 93). The results of these analyses are shown in Additional file 1: Table S3 using a genotype test for trend with a threshold of *P*_*trend*_ < 0.05. Variants with a MAF of less than 0.05 or a HWE *P* value of less than 1 × 10^−5^ were removed from this analysis. No variants retained significance after adjustment for multiple comparisons.

## Discussion

This study demonstrated the successful application of a customised vitamin D targeted NGS panel in two renal transplant cohorts. The library preparation procedure successfully enriched for the targeted genomic regions designed using the Ion AmpliSeq™ designer tool. Sequencing runs provided sufficient sequencing quality, data output and validated the effective library preparation and panel design. The panel achieved sufficient (>30 fold) coverage to reliably identify variants associated with vitamin D metabolism and regulation. The representative coverage maps shown in Fig. 4 demonstrated that similar coverage patterns were obtained using both sequencers which confirms the consistency of the targeted panel.

The Ion Torrent Suite™ indicated that the overall quality of the data obtained from sequencing runs on the Ion S5™ XL was better than the data quality from sequencing performed on the Ion PGM™. For example, the bead loading onto the silicon chips was higher in runs performed on the Ion S5™ XL as shown in Figs. 5, 6 and 7. Higher levels of data output were achieved when the panel was used on the Ion S5™ XL rather than the Ion PGM™ as shown by the sequencing statistics in Table 2. These differences are almost certainly due to the chip type employed during the sequencing runs. The Ion 530™ chip used in this study has an inherently higher capacity (~38 million wells per chip) than the Ion 316™ (~6 million wells per chip) or Ion 318™ chips (~11 million wells per chip). Higher chip capacity allows more samples to be included on the same chip with higher coverage for each sample. The number of samples and coverage level can be balanced when planning a sequencing run to achieve optimum results.

In addition to larger sample capacity and higher coverage potential on the Ion S5™ XL, this sequencer was demonstrated to identify variants correctly more often than the Ion PGM™. When variants called differently on both sequencers were investigated by Sanger sequencing, it was determined that the Ion S5™ XL was correct in 14 instances while the Ion PGM was correct nine times. The Ion S5™XL did call slightly higher numbers of correct variants than the Ion PGM™. Despite the differences between the sequencers, the majority of reported results were consistent between both machines which is evidence that this custom panel is effective and reliable.

Association analyses were conducted to demonstrate the efficacy and potential applications of the custom NGS panel. These analyses were conducted in two kidney transplant cohorts to investigate the association between the targeted variants and either changes in vitamin D levels post-transplant or the development of NODAT, a serious post-transplant complication. None of the variants identified in the presented analyses reached genome-wide significance thresholds due to the small number of samples included. This was expected as these analyses were designed to provide proof-of-concept and were not intended to represent a well powered genotype association study. Hence, no adjustments for multiple testing have been made in the presented results.

The Belfast renal transplant cohort was used to investigate the association of NODAT with these vitamin D related regions on the custom panel. The results presented in Additional file 1: Table S3 show the nominal association of the NODAT phenotype at the *P*_*trend*_ < 0.05 threshold in a genotype association test for trend.

**Fig. 10:**
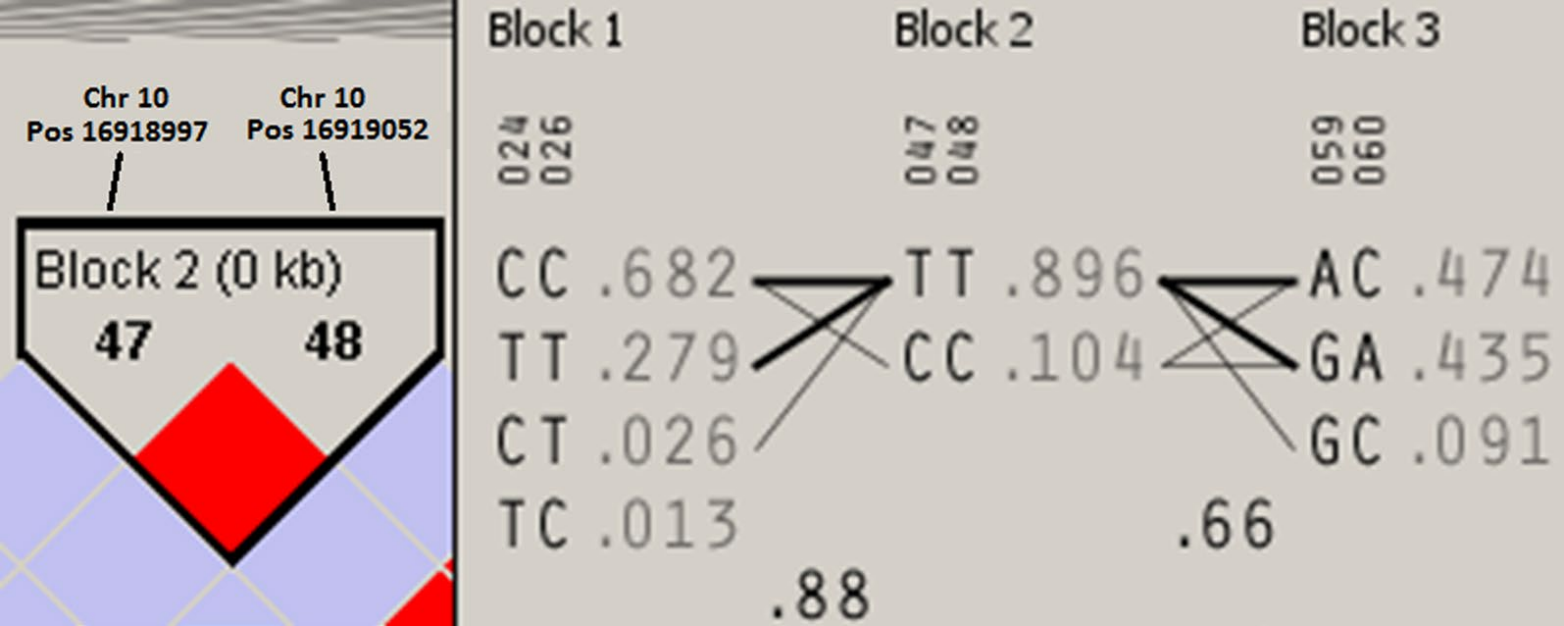
Haploview™ image of SNPs rs1801240 and rs1801239. This image shows that these two missense mutations identified by the Ion S5™ XL are tightly linked (strong linkage disequilibrium is indicated by the *red* diamond)

Two missense mutations (rs1801240 and rs1801239) in the *CUBN* gene exon 57 were nominally associated with NODAT following this analysis. These mutations are in strong linkage disequilibrium as shown by the Haploview image in Fig. 10 (D’ = 1.0, 95% CI 0.89–1.0). An article by Tzur and colleagues has previously described a haplotype characterised by *CUBN* exon 57 and 42 SNPs including these variants [49]. Tzur associated this haplotype with an increased incidence of albuminuria [49]. Proteinuria post-transplant is a known risk factor for NODAT and if the patients were predisposed to albuminuria this may have in part contributed to the development of the NODAT transplant complication. A subset of the Birmingham renal transplant cohort was used to replicate these analyses (Additional file 1: Table S2). Notably, rs1801239 was also identified in this cohort. The variant rs1801239 had a similar direction of effect following logistic regression in the previous GWAS (OR: 3.904; *P*_*LR*_: 0.001; 95% CI 1.7–8.8). It is important to note that none of the highest ranking variants identified in this analysis association analysis were identified in a previous genome wide association study (GWAS) conducted in the same Northern Irish renal transplant population [46]. In addition, none of variants in the exon 57 and 42 regions of the *CUBN* gene covered on the GWAS panel were associated with NODAT in this previous study at the genome-wide significance threshold.

In the Birmingham renal transplant cohort, the association between change in vitamin D levels after kidney transplantation and the variants on the custom panel was investigated. The results of these analyses are shown in Additional file 1: Table S3. Three SNPs were found in common between the analyses of vitamin D change at 3 and 12 months including two variants in the promoter rank region of *CYP27B1*.

The seasonality of testing for vitamin D levels was not considered during this study. This is a potential limitation, but this study involved renal transplant recipients who are known to have an inherently higher risk of skin malignancy associated with immunosuppression and are therefore advised to use high factor sunscreens and to stay out of direct sunlight [50, 51]. There clinical factors would be likely to reduce the impact of seasonality on the vitamin D levels in these individuals.

Most of the associated variants, using data from both sequencers and in both cohorts, were in the cubilin (*CUBN*) gene. This gene encodes the peripheral membrane protein cubilin which contributes to the uptake of vitamin B12 and metabolism of vitamin D [52]. A previous study demonstrated that patients with non-functioning cubilin lose significant amounts of 25-hydroxyvitamin D3 in their urine, reducing their plasma 1,25-dihydroxyvitamin D levels [22]. Cubilin is essential for the reabsorption of proteins in the proximal tubules of the kidney [53]. A study published in 2012 investigated the association between variants in the *CUBN* gene and graft failure in renal transplant recipients [54] following a GWAS which linked these variants to albuminuria [55, 56]. The study found that the *CUBN* variant rs7918972 in the organ donor genotype was associated with graft failure in the transplant recipient population. It is important to note that the *CUBN* gene is large (0.31 mega-bases) and so it is possible that the large number of variants identified in this gene in the presented study may be a result of the size of the gene rather than a of true association with the investigated phenotype. Larger genes will naturally harbour more variants than smaller genes included on the panel. It is important to account for this by applying *P* value threshold which accounts for multiple testing in further well powered studies [57].

The association analysis performed on these cohorts using data from two different sequencers demonstrates the capacity of the custom panel to detect SNP associations. To gain more statistically significant and meaningful associations the panel should be applied to considerably larger cohorts. This NGS panel will allow collaborating laboratories to perform identical targeted sequencing for vitamin D associated genomic regions. Studies that demonstrate significant associations between the phenotype of interest because of investigations using this targeted panel, will require further analysis using functional studies to explore the mechanisms underpinning the associations.

Sanger sequencing is costly when focusing on many variants or genes such as those targeted in this panel. Sanger sequencing is also more time consuming than NGS techniques which offer a higher throughput approach to variant detection. Targeted NGS panels such as this custom AmpliSeq™ panel offer an attractive alternative to Sanger sequencing for projects encompassing larger numbers of target variants. Whole exome sequencing is a popular alternative to custom NGS panels but this approach is more expensive than targeted panels. The larger volume of sequencing data produced by exome sequencing requires more storage space and bioinformatic analysis which can be associated with significant costs. Incidental findings are also a more significant issue when using whole exome sequencing.

This NGS panel represents a cost effective, fast molecular test with minimal hands on time. This high throughput panel can be used to sequence one pool of 96 amplicons on a single Ion 318™ chip. The Belfast renal transplant cohort in contrast has more limited genetic heterogeneity with a very tightly defined NODAT phenotype. It is vital to note that the association results presented in this study are primarily demonstrating proof-of-principle that this NGS panel can be effectively applied to a range of varying populations rather than trying to establish definitive evidence of associations. The patient numbers included in these cohorts do not provide enough power to reliably identify associations with small to moderate effect sizes, but do confirm the utility of the panel. Despite these limitations, this reliable and cost effective vitamin D panel was successfully used to sequence the targeted vitamin D regions in two renal transplant populations with excellent coverage and successful variant calling.

Homo-polymer regions are one of the limiting factors in any project relying on Ion Torrent™ sequencers which tend to underestimate or overestimate the length of repeated, identical base calls. A previous study reported that 94–97% of total error on the Ion PGM™ was due to homo-polymer regions [58]. During this study, these regions were indeed shown to have a high error rate. Most of the discrepancy variants investigated by Sanger sequencing were close to these repetitive regions, such as the DNA fragment shown in Fig. 6. The design of the panel itself was also shaped by these homo-polymer regions. Table 1 shows that some regions could not be covered by the panel due to repetitive regions in the target sequences.

## Conclusions

During the course of this study, a custom NGS panel was designed, implemented and validated. Two separate kidney transplant cohorts were used to demonstrate the potential of the panel and to verify the effectiveness of this targeted NGS approach. The custom panel successfully targeted genomic regions putatively involved in vitamin D regulation.

A comparison of the targeted panel using the Ion PGM™ and Ion S5™ XL sequencers from the Ion Torrent range was performed during this study. This was achieved by comparing the variants identified by the Ion Torrent™ variant caller plug-in following runs of the same panel on the two sequencers. The results of this analysis showed that the Ion S5™ XL identified more variants, with higher accuracy than the Ion PGM™ in comparable runs. Therefore, although the panel was effective on both sequencers, the inherent advantages of the Ion S5™ XL provided clear improvements in performance over the Ion PGM™.

Proof-of-concept association analyses were performed using the sequencing data obtained using the custom NGS panel. It is possible that the mutations in vitamin D linked to change in vitamin D levels after kidney transplantation or to NODAT in these analyses are valuable. However, the aim of the analysis was to demonstrate the use of the custom NGS panel rather than undertaking comprehensive associations with gene variants in these cohorts. In order to further elucidate the link between these variants and the phenotypes investigated here, larger scale association studies are necessary alongside functional studies, particularly as change in BMI was included in the covariate model. This work could lead to further understanding of the pathways involved in the development of NODAT for example, leading to treatments and perhaps diet and lifestyle recommendations which are better tailored to individuals with certain genetic profiles.

Overall, this study has demonstrated the effectiveness of this novel, targeted NGS panel for genomic regions putatively involved in vitamin D regulation and metabolism. This panel has a wide range of potential applications and will be valuable to an assortment of future projects.

## Additional file

**Additional file 1: Table S1.** Belfast cohort NODAT association results from Ion S5™ XL and Ion PGM™ sequencing data (Inf= infinity). **Table S2.** Association of NODAT phenotype in Birmingham cohort (Inf=infinity). **Table S3.** Birmingham association results from Ion PGM™ sequencing data.

## Abbreviations

BMI: body mass index
CKD: chronic kidney disease
GWAS: genome-wide association study
ISP: ion sphere particle
NGS: next generation sequencing
NODAT: new onset diabetes after transplantation
OGTT: oral glucose tolerance test
PGM: personal genome machine
RXR: retinoid X receptor
TBP: TATA binding protein
TF: transcription factors
UVB: ultraviolet B
VDR: vitamin D receptor
VDRE: vitamin D response element

## References

[1] Kimball SM, Hanwell HE (2012). Calcitriol” is not synonymous with “vitamin D. Mult Scler Int.

[2] Powers JG, Gilchrest BA (2012). What you and your patients need to know about vitamin D. Semin Cutan Med Surg.

[3] Hathcock JN, Shao A, Vieth R, Heaney R (2007). Risk assessment for vitamin D. Am J Clin Nutr.

[4] Wood CL, Cheetham TD (2016). Vitamin D: increasing supplement use among at-risk groups (NICE guideline PH56). Arch Dis Childhood-E.

[5] Bailey R, Cooper JD, Zeitels L, Smyth DJ, Yang JH, Walker NM (2007). Association of the vitamin D metabolism gene CYP27B1 with type 1 diabetes. Diabetes.

[6] Christakos S, Dhawan P, Verstuyf A, Verlinden L, Carmeliet G (2016). Vitamin D: metabolism, molecular mechanism of action, and pleiotropic effects. Physiol Rev.

[7] Wang TJ, Zhang F, Richards JB, Kestenbaum B, van Meurs JB, Berry D (2010). Common genetic determinants of vitamin D insufficiency: a genome-wide association study. Lancet.

[8] Biggar PH, Liangos O, Fey H, Brandenburg VM, Ketteler M (2011). Vitamin D, chronic kidney disease and survival: a pluripotent hormone or just another bone drug?. Pediatr Nephrol.

[9] Haussler MR, Whitfield GK, Kaneko I, Haussler CA, Hsieh D, Hsieh JC (2013). Molecular mechanisms of vitamin D action. Calcif Tissue Int.

[10] Pike JW, Meyer MB, Bishop KA (2012). Regulation of target gene expression by the vitamin D receptor - an update on mechanisms. Rev Endocr Metab Disord.

[11] Fetahu IS, Höbaus J, Kállay E (2014). Vitamin D and the epigenome. Front Physiol.

[12] Shroff R, Wan M, Rees L (2012). Can vitamin D slow down the progression of chronic kidney disease?. Pediatr Nephrol.

[13] Ramagopalan SV, Heger A, Berlanga AJ, Maugeri NJ, Lincoln MR, Burrell A (2010). A ChIP-seq defined genome-wide map of vitamin D receptor binding: associations with disease and evolution. Genome Res.

[14] Martin RJL, McKnight AJ, Patterson CC, Sadlier DM, Maxwell AP, WUGS Grp (2010). A rare haplotype of the vitamin D receptor gene is protective against diabetic nephropathy. Nephrol Dial Transplant.

[15] Chang CK, Mulholland HG, Cantwell MM, Anderson LA, Johnston BT, McKnight AJ (2012). Vitamin D receptor gene variants and esophageal adenocarcinoma risk: a population-based case-control study. J Gastrointest Cancer.

[16] Deschasaux M, Souberbielle JC, Latino-Martel P, Sutton A, Chamaux N, Druesne-Pecollo N (2015). Prospective associations between vitamin D status, vitamin D-related gene polymorphisms, and risk of tobacco-related cancers. Am J Clin Nutr.

[17] Reimers LL, Crew KD, Bradshaw PT, Santella RM, Steck SE, Sirosh I (2015). Vitamin D-related gene polymorphisms, plasma 25-hydroxyvitamin D, and breast cancer risk. Cancer Cause Control.

[18] Arem H, Yu K, Xiong XQ, Moy K, Freedman ND, Mayne ST (2015). Vitamin D metabolic pathway genes and pancreatic cancer risk. PloS ONE.

[19] Waterhouse M, Tran B, Armstrong BK, Baxter C, Ebeling PR, English DR (2014). Environmental, personal, and genetic determinants of response to vitamin D supplementation in older adults. J Clin Endocrinol Metab.

[20] Hansen JG, Gao W, Dupuis J, O’Connor GT, Tang W, Kowgier M (2015). Association of 25-Hydroxyvitamin D status and genetic variation in the vitamin D metabolic pathway with FEV1 in the Framingham Heart Study. Respir Res.

[21] Wise LA, Ruiz-Narvaez EA, Haddad SA, Rosenberg L, Palmer JR (2014). Polymorphisms in vitamin D-related genes and risk of uterine leiomyomata. Fertil Steril.

[22] Ramos-Lopez E, Lange B, Penna-Martinez M, Bruck P, Swiech K, Mauf S (2010). The role of cubilin gene polymorphisms and their influence on 25(OH)D-3 and 1,25(OH)(2)D-3 plasma levels in type 1 diabetes patients. J Steroid Biochem.

[23] Padmanabhan S (2014). Handbook of pharmacogenomics and stratified medicine.

[24] Mertes F, Elsharawy A, Sauer S, van Helvoort JM, van der Zaag PJ, Franke A (2011). Targeted enrichment of genomic DNA regions for next-generation sequencing. Brief Funct Genom.

[25] Samorodnitsky E, Jewell BM, Hagopian R, Miya J, Wing MR, Lyon E (2015). Evaluation of hybridization capture versus amplicon-based methods for whole-exome sequencing. Hum Mutat.

[26] Liu L, Li Y, Li S, Hu N, He Y, Pong R (2012). Comparison of next-generation sequencing systems. J Biomed Biotechnol.

[27] Gracia-Iguacel C, Gallar P, Qureshi AR, Ortega O, Mon C, Ortiz M (2010). Vitamin D deficiency in dialysis patients: effect of dialysis modality and implications on outcome. J Renal Nutr.

[28] Razzaque MS (2013). Phosphate and vitamin D in chronic kidney disease preface. Contrib Nephrol.

[29] Kaptein EM, Yi SS, Endres DB, Kaptein JS, Chan LS (2014). Serum 25-hydroxyvitamin D deficiency: a risk factor for chronic kidney disease in ambulatory indigent patients. Endocr Pract.

[30] Pilz S, Iodice S, Zittermann A, Grant WB, Gandini S (2011). Vitamin D status and mortality risk in CKD: a meta-analysis of prospective studies. Am J Kidney Dis.

[31] Santoro D, Pellicano V, Cernaro V, Lacava V, Lacquaniti A, Atteritano M (2016). Role of vitamin D in vascular complications and vascular access outcome in patients with Chronic kidney disease. Curr Med Chem.

[32] McGregor R, Li G, Penny H, Lombardi G, Afzali B, Goldsmith DJ (2014). Vitamin D in renal transplantation-from biological mechanisms to clinical benefits. Am J Transplant Off J Am Soc Transplant Am Soc Transplant Surg.

[33] Eyal O, Aharon M, Safadi R, Elhalel MD (2013). Serum vitamin D levels in kidney transplant recipients: the importance of an immunosuppression regimen and sun exposure. Isr Med Assoc J.

[34] Stavroulopoulos A, Cassidy MJ, Porter CJ, Hosking DJ, Roe SD (2007). Vitamin D status in renal transplant recipients. Am J Transplant Off J Am Soc Transplant Am Soc Transplant Surg.

[35] Le Fur A, Fournier MC, Gillaizeau F, Masson D, Giral M, Cariou B (2016). Vitamin D deficiency is an independent risk factor for PTDM after kidney transplantation. Transpl Int.

[36] Yao B, Chen X, Shen FX, Xu W, Dong TT, Chen LZ (2013). The incidence of posttransplantation diabetes mellitus during follow-up in kidney transplant recipients and relationship to Fok1 vitamin D receptor polymorphism. Transpl Proc.

[37] Courbebaisse M, Alberti C, Colas S, Prie D, Souberbielle JC, Treluyer JM (2014). VITamin D supplementation in renAL transplant recipients (VITALE): a prospective, multicentre, double-blind, randomized trial of vitamin D estimating the benefit and safety of vitamin D-3 treatment at a dose of 100,000 UI compared with a dose of 12,000 UI in renal transplant recipients: study protocol for a double-blind, randomized, controlled trial. Trials.

[38] Keyzer CA, Riphagen IJ, Joosten MM, Navis G, Muller Kobold AC, Kema IP (2015). Associations of 25(OH) and 1,25(OH)2 vitamin D with long-term outcomes in stable renal transplant recipients. J Clin Endocrinol Metab.

[39] Chand S, McKnight AJ, Shabir S, Chan W, McCaughan JA, Maxwell AP (2016). Analysis of single nucleotide polymorphisms implicate mTOR signalling in the development of new-onset diabetes after transplantation. Biochimica et Biophysica Acta Clinical.

[40] Collins AJ, Foley RN, Chavers B, Gilbertson D, Herzog C, Johansen K (2012). ‘United States renal data system 2011 annual data report: Atlas of chronic kidney disease & end-stage renal disease in the United States. Am J Kidney Dis.

[41] Cooper JD, Smyth DJ, Walker NM, Stevens H, Burren OS, Wallace C (2011). Inherited variation in vitamin D genes is associated with predisposition to autoimmune disease type 1 diabetes. Diabetes.

[42] Nejentsev S, Godfrey L, Snook H, Rance H, Nutland S, Walker NM (2004). Comparative high-resolution analysis of linkage disequilibrium and tag single nucleotide polymorphisms between populations in the vitamin D receptor gene. Hum Mol Genet.

[43] Gaynor JJ, Ciancio G, Guerra G, Sageshima J, Hanson L, Roth D (2015). Multivariable risk of developing new onset diabetes after transplant-results from a single-center study of 481 adult, primary kidney transplant recipients. Clin Transplant.

[44] Pham PT, Pham PM, Pham SV, Pham PA, Pham PC (2011). New onset diabetes after transplantation (NODAT): an overview. Diabetes Metab Syndr Obes.

[45] Kim Y, Kim JR, Choi H, Hwang JW, Jang HR, Lee JE (2013). Patients with persistent new-onset diabetes after transplantation have greater weight gain after kidney transplantation. J Korean Med Sci.

[46] McCaughan JA, McKnight AJ, Maxwell AP (2014). Genetics of new-onset diabetes after transplantation. J Am Soc Nephrol.

[47] Flicek P, Amode MR, Barrell D, Beal K, Billis K, Brent S (2014). Ensembl 2014. Nucleic Acids Research..

[48] Cullen M, Boland JF, Schiffman M, Zhang X, Wentzensen N, Yang Q (2015). Deep sequencing of HPV16 genomes: a new high-throughput tool for exploring the carcinogenicity and natural history of HPV16 infection. Papillomavirus Res.

[49] Tzur S, Wasser WG, Rosset S, Skorecki K (2012). Linkage disequilibrium analysis reveals an albuminuria risk haplotype containing three missense mutations in the cubilin gene with striking differences among European and African ancestry populations. BMC Nephrol.

[50] Tepeoglu M, Ayva S, Ok Atilgan A, Tunca MZ, Ozdemir BH, Moray G (2014). Nonmelanoma skin cancer after kidney transplant. Exp Clin Transplant.

[51] Bannon FJ, McCaughan JA, Traynor C, O’Brien K, Gavin AT, Maxwell AP (2014). Surveillance of nonmelanoma skin cancer incidence rates in kidney transplant recipients in Ireland. Transplantation.

[52] Nykjaer A, Fyfe JC, Kozyraki R, Leheste JR, Jacobsen C, Nielsen MS (2001). Cubilin dysfunction causes abnormal metabolism of the steroid hormone 25(OH) vitamin D(3). Proc Natl Acad Sci.

[53] Christensen EI, Nielsen R (2007). Role of megalin and cubilin in renal physiology and pathophysiology. Rev Physiol Biochem Pharmacol.

[54] Reznichenko A, Snieder H, van den Born J, de Borst MH, Damman J, van Dijk MC (2012). CUBN as a novel locus for end-stage renal disease: insights from renal transplantation. PLoS ONE.

[55] Boger CA, Chen MH, Tin A, Olden M, Kottgen A, de Boer IH (2011). CUBN is a gene locus for albuminuria. J Am Soc Nephrol JASN.

[56] Christensen EI, Nielsen R, Birn H (2013). From bowel to kidneys: the role of cubilin in physiology and disease. Nephrol Dial Transplant.

[57] Sham PC, Purcell SM (2014). Statistical power and significance testing in large-scale genetic studies. Nat Rev Genet.

[58] Bragg LM, Stone G, Butler MK, Hugenholtz P, Tyson GW (2013). Shining a light on dark sequencing: characterising errors in Ion Torrent PGM data. PLoS Comput Biol.

