# Supplementary material for: Design and implementation of a custom next generation sequencing panel for selected vitamin D associated genes
Source: BMC Res Notes. 2017 Jul 28;10:348. doi: 10.1186/s13104-017-2664-z (PMC5534126; doi:10.1186/s13104-017-2664-z)
Supplement: Supplementary file 1 — Additional file 1: Table S1. Belfast cohort NODAT association results from Ion S5™ XL and Ion PGM™ sequencing data (Inf= infinity). Table S2. Association of NODAT phenotype in Birmingham cohort (Inf=infinity). Table S3. Birmingham association results from Ion PGM™ sequencing data. [file 13104_2017_2664_MOESM1_ESM.docx]

**Table S1: Belfast cohort NODAT association results from Ion S5™ XL and Ion PGM™ sequencing data (Inf= infinity)**

| **Chr** | **Location** | **rs number** | **MAF** | **HWE** | **Gene** | **Functional Effect** | ***P_trend_*** | **Logistic Regression** | | |
| --- | --- | --- | --- | --- | --- | --- | --- | --- | --- | --- |
|  |  |  |  | ***P value*** |  |  |  | ***P_LR_*** | **OR** | **95% CI** |
| 11 | 71185621 | Novel | 0.1091 | 1 | *NADSYN1* | Intron | 0.001713 | 0.01541 | 0.05737 | 0.006-0.579 |
| 12 | 48276424 | Novel | 0.3312 | 3.73E-06 | *VDR* | Intron | 0.01227 | 0.02858 | 0.1662 | 0.033-0.829 |
| 12 | 48251533 | rs11168266 | 0.474 | 0.8194 | *VDR* | Intron | 0.01347 | 0.008257 | 0.2454 | 0.087-0.696 |
| 10 | 16967586 | [rs62619939](http://grch37.ensembl.org/Homo_sapiens/Variation/Explore?db=core;r=10:16967586-16967586;source=dbSNP;v=rs62619939;vdb=variation;vf=115633130) | 0.1169 | 1 | *CUBN* | Missense | 0.01652 | 0.0871 | 6.286 | 0.765-51.64 |
| 10 | 16918997 | rs1801240 | 0.1039 | 1 | *CUBN* | Missense | 0.02858 | 0.2225 | 2.814 | 0.534-14.84 |
| 10 | 16919052 | rs1801239 | 0.1039 | 1 | *CUBN* | Missense | 0.02858 | 0.2225 | 2.814 | 0.534-14.84 |
| 10 | 17110297 | rs10904871 | 0.01948 | 1 | *CUBN* | Intron | 0.04134 | 0.9987 | 1.96x10^-10^ | 0-inf |
| 10 | 17110598 | rs78549445 | 0.01948 | 1 | *CUBN* | Intron | 0.04134 | 0.9987 | 1.96x10^-10^ | 0-inf |
| 10 | 17127826 | rs3740163 | 0.01948 | 1 | *CUBN* | Intron | 0.04134 | 0.9987 | 1.96x10^-10^ | 0-inf |
| 12 | 48238837 | rs7975232 | 0.4416 | 0.4876 | *VDR* | Intron | 0.04222 | 0.02011 | 0.3058 | 0.113-0.831 |
| 12 | 58157281 | rs4646537 | 0.03247 | 1 | *CYP27B1* | Intron | 0.04522 | 0.9985 | 7.6x10^8^ | 0-inf |

**Table S2: Association of NODAT phenotype in Birmingham cohort (Inf=infinity)**

| **Chr** | **Location** | **rs number** | **MAF** | **HWE** | **Gene** | **Functional Effect** | ***P_trend_*** | **Logistic Regression** | | |
| --- | --- | --- | --- | --- | --- | --- | --- | --- | --- | --- |
|  |  |  |  | ***P value*** |  |  |  | ***P_LR_*** | **OR** | **95% CI** |
| 12 | 48278010 | rs1989969 | 0.364 | 0.434 | *VDR* | Intron | 0.012 | 0.007 | 0.170 | 0.0473-0.612 |
| 11 | 71169547 | rs2276360 | 0.371 | 1 | *NADSYN1* | Missense | 0.013 | 0.029 | 0.285 | 0.0926-0.8774 |
| 12 | 58160738 | novel | 0.144 | 0.570 | *CYP27B1* | Promoter Flank | 0.021 | 0.020 | 12.600 | 1.490-106.5 |
| 12 | 48337069 | rs4073729 | 0.100 | 1 | *VDR* | Upstream Variant | 0.025 | 0.999 | 2.61x10^18^ | 0-inf |
| 10 | 16919052 | rs1801239 | 0.129 | 0.276 | *CUBN* | Missense | 0.026 | 0.023 | 4.194 | 1.219-14.42 |
| 11 | 71184498 | rs2276358 | 0.279 | 0.759 | *NADSYN1* | Intron | 0.035 | 0.062 | 0.305 | 0.0872-1.064 |
| 12 | 48284968 | rs3922882 | 0.273 | 0.536 | *VDR* | Intron | 0.038 | 0.039 | 0.250 | 0.0670-0.933 |
| 11 | 71183474 | rs2282621 | 0.364 | 0.795 | *NADSYN1* | Intron | 0.045 | 0.092 | 0.395 | 0.134-1.164 |
| 11 | 71183477 | rs2282620 | 0.364 | 0.795 | *NADSYN1* | Intron | 0.045 | 0.092 | 0.395 | 0.134-1.164 |

**Table S3: Birmingham association results from Ion PGM™ sequencing data**

| **Chr** | **Location** | **rs Number** | **MAF** | **HWE** | **Gene Symbol** | **Functional Effect** | ***P_trend_*** | **Logistic Regression** | | |
| --- | --- | --- | --- | --- | --- | --- | --- | --- | --- | --- |
|  |  |  |  |  |  |  |  | ***P_LR_*** | **Beta Value** | **SE** |
| **Change after 12 months** | | | | | | | | | | |
| 11 | 71192313 | rs57500217 | 0.070 | 0.337 | *NADSYN1* | Non-coding exon | 0.001 | 0.873 | 5.645 | 35.010 |
| 11 | 71192366 | rs59487812 | 0.070 | 0.337 | *NADSYN1* | Non-coding exon | 0.001 | 0.157 | 0.779 | 0.543 |
| 11 | 71192439 | rs59379414 | 0.076 | 0.387 | *NADSYN1* | Stop-gain | 0.001 | 1.75x10^-10^ | 327.100 | 41.860 |
| 10 | 17024503 | rs1801231 | 0.145 | 0.013 | *CUBN* | Missense | 0.001 |  |  |  |
| 10 | 17026010 | rs2291523 | 0.064 | 1.000 | *CUBN* | Intron | 0.006 |  |  |  |
| 10 | 17024615 | rs1801229 | 0.267 | 0.782 | *CUBN* | Synonymous | 0.008 |  |  |  |
| 11 | 14913575 | rs12794714 | 0.338 | 0.706 | *CYP2R1* | Synonymous | 0.013 | 0.608 | -15.680 | 30.340 |
| 12 | 58160738 | novel | 0.131 | 0.579 | *CYP27B1* | Promoter flank | 0.015 |  |  |  |
| 12 | 48285414 | rs11168287 | 0.443 | 0.608 | *VDR* | Intron | 0.019 | 0.064 | 0.764 | 0.400 |
| 12 | 48366550 | rs2071356 | 0.279 | 1.000 | *VDR* | Downstream | 0.020 | 0.053 | -0.957 | 0.484 |
| 20 | 52770709 | novel | 0.058 | 1.000 | *CYP24A1* | Upstream | 0.020 |  |  |  |
| 12 | 58160737 | novel | 0.205 | 0.056 | *CYP27B1* | Promoter flank | 0.021 |  |  |  |
| 10 | 16911605 | rs703075 | 0.384 | 0.503 | *CUBN* | Intron | 0.021 | 0.592 | 9.277 | 17.210 |
| 12 | 58160735 | novel | 0.189 | 0.103 | *CYP27B1* | Promoter flank | 0.028 | 0.069 | 0.749 | 0.398 |
| 12 | 58160736 | novel | 0.189 | 0.103 | *CYP27B1* | Promoter flank | 0.028 | 0.150 | -0.424 | 0.288 |
| 10 | 16989266 | novel | 0.174 | 0.064 | *CUBN* | Exon | 0.034 | 0.110 | 0.948 | 0.582 |
| 12 | 48272895 | rs2228570 | 0.393 | 0.592 | *VDR* | Start-loss | 0.035 |  |  |  |
| **Change after 3 months** | | | | | | | | | | |
| 4 | 72618334 | rs7041 | 0.488 | 1.000 | *GC* | Missense | 0.004 | 0.935 | -0.634 | 7.737 |
| 11 | 14913575 | rs12794714 | 0.338 | 0.706 | *CYP2R1* | Synonymous | 0.005 | 0.478 | -13.250 | 18.560 |
| 12 | 58160735 | novel | 0.189 | 0.103 | *CYP27B1* | Promoter flank | 0.008 | 0.073 | 0.793 | 0.431 |
| 12 | 58160736 | novel | 0.189 | 0.103 | *CYP27B1* | Promoter flank | 0.008 | 0.398 | -0.261 | 0.305 |
| 12 | 48337069 | rs4073729 | 0.167 | 1.000 | *VDR* | Upstream | 0.008 | 0.405 | -0.257 | 0.305 |
| 10 | 16911605 | rs703075 | 0.384 | 0.503 | *CUBN* | Intron | 0.009 | 0.488 | 7.088 | 10.160 |
